# The Role of Race and Gender in Nutrition Habits and Self-Efficacy: Results from the Young Adult Weight Loss Study

**DOI:** 10.1155/2017/5980698

**Published:** 2017-04-13

**Authors:** Janna D. Stephens, Andrew Althouse, Alai Tan, Bernadette Mazurek Melnyk

**Affiliations:** ^1^The Ohio State University College of Nursing, Columbus, OH, USA; ^2^Heart and Vascular Institute, University of Pittsburgh Medical Center, Pittsburgh, PA, USA

## Abstract

Overweight and obesity are a massive public health problem and young adults are at high risk for gaining weight once they enter a college. This study sought to examine gender and race as they relate to nutrition habits and self-efficacy in a population of diverse young adults from the Young Adult Weight Loss Study. Participants (*N* = 62) were 29% males, 38.7% white, 33.8% Asian, and 12.9% African American. Males had lower self-efficacy for healthy eating (mean score = 92.5, SD = 17.1) compared to females (mean = 102.3, SD = 13.7, *p* = 0.02). Males had higher consumption of sodium compared to females (4308 versus 3239 milligrams/day, *p* = 0.01). There were no significant differences across racial subgroups in self-efficacy for healthy eating (*p* = 0.67) or self-efficacy for exercise (*p* = 0.61). Higher self-efficacy scores for healthy eating were significantly associated with less total sodium (*r* = −0.37, *p* = 0.007), greater fruit consumption, and less saturated fat. Our results indicate that weight loss interventions should be individualized and that there may be specific areas to target that are different for men and women. Additional larger studies should be conducted to confirm if racial differences exist across nutrition habits and self-efficacy and to confirm gender differences noted in this study.

## 1. Introduction

Overweight/obesity is a major public health concern in the United States (US) as reported by the most recent National Health and Nutrition Examination Survey (NHANES), with 60.3% of adults aged 20–39 years being overweight (BMI 25.0–29.9) or obese (BMI ≥ 30) [1]. Certain subgroups of the population have higher rates of overweight/obesity; for example, non-Hispanic blacks are reported to have a rate of 71.9% among those aged 20–39 years [1]. Obesity has been linked to many significant health concerns, including hypertension, type 2 diabetes, coronary heart disease, stroke, osteoarthritis, certain types of cancer, and mortality [2, 3].

The transition out of high school to college or an independent setting has been identified as a critical time for establishing healthy lifestyle habits as individuals are traditionally more independent and they experience major changes in their overall habits, including eating and exercise [3–8]. A recent report from the American College Health Association indicated that less than 5% of college students consume 5 or more servings of fruit/vegetables per day and 80% report not meeting the Physical Activity Guidelines for Americans [9]. In addition, this newfound independence can lead to a lack of self-efficacy for healthy eating and exercise [6, 10, 11]. Being overweight/obese during these transition years seriously increases the probability that these youth will remain overweight.

We sought to characterize factors associated with self-efficacy for healthy eating and self-efficacy for exercise in a group of young adults from the Young Adult Weight Loss (YAWL) Study.

## 2. Methods

### 2.1. Study Design

This descriptive study used baseline data collected from the YAWL randomized controlled trial testing a weight loss intervention in young adults who were overweight or obese [12]. The full details and methods of the YAWL randomized controlled trial are described elsewhere [12]. For the purposes of this study, we examined the distribution and correlations of specific baseline data collected including healthy eating self-efficacy, exercise self-efficacy, nutrition knowledge, exercise habits, and healthy eating habits.

### 2.2. Sample and Setting

In the YAWL study those who met the inclusion criteria (BMI 25–40 kg/m^2^, aged 18–25 years, and owning an iPhone or Android device) were eligible to participate. Participants were recruited in and around a university setting on the east coast, although participants were not required to be a college or university student. Recruitment methods consisted of flyers/posters, social media posts, email announcements, and word of mouth. Sixty-two participants consented to be in the study. For the purposes of this study, all 62 individuals who had baseline data were included in the analysis.

### 2.3. Measures and Instruments

Data was collected at the baseline study visit and entered into the Research Electronic Data Capture (REDCap) application, a secure, web-based application. Height and weight were measured by research personnel with the participant in light clothing. All questionnaires were collected on paper and pencil, except the Automated Self-Administered 24-Hour Recall (ASA24), which was collected via the participant's computer immediately following the baseline visit. Physical activity was assessed using the Godin Leisure-Time Exercise Questionnaire, dietary habits were assessed using the ASA-24, nutritional knowledge was measured with the nutrition knowledge survey, and self-efficacy was measured for both healthy eating and physical activity.

The Godin Leisure-Time Exercise Questionnaire is self-administered and assesses a total score for strenuous, moderate, and mild activity over a 7-day period as well as assessing the time spent in activity long enough to work up a sweat [13]. The ASA24 is a web-based, automated self-administered 24-hour recall of foods. The ASA24 provides analysis on calories, nutrients, and food group estimates [14]. Information obtained from the ASA-24 included caloric intake, food pyramid equivalents, and nutrients from all foods reported according to the Food and Nutrient Database for Dietary Studies [14]. For the purposes of this study, we examined total fiber, sodium, saturated fat, fruit servings, and vegetable servings per day.

The Self-Efficacy for Healthy Eating Scale was a 13-item questionnaire that explored a person's belief in their ability to make better food choices in given situations. The Self-Efficacy for Exercise Scale was a 14-item questionnaire assessing an individual's belief in their ability to perform physical activity in given situations. Scores were calculated by adding each item score together to achieve an overall total score. The nutrition knowledge survey assesses knowledge on food consumption from sodium to fat to overall healthy meals [15]. This survey was scored by adding the numbers that were correct together to achieve a total score out of a maximum 40 points.

### 2.4. Statistical Analysis

Descriptive statistics were used to summarize sample characteristics and baseline measures of healthy eating self-efficacy, exercise self-efficacy, nutrition knowledge, exercise habits, and healthy eating habits by gender and racial groups. *t*-test and analysis of variance were used to test whether there was significant between-group difference in these baseline measures. Pearson correlation coefficients were used to examine the pair-wise associations between BMI, healthy eating self-efficacy, exercise self-efficacy, nutrition knowledge, exercise habits, and healthy eating habits. All significance tests were two-sided with significant level at 0.05. We used SAS 9.4 (SAS Institute®, Cary, NC) for the analysis.

## 3. Results

Participants (*N* = 62) were 18–25 years old, 29% males, 38.7% white, 33.8% Asian, and 12.9% African American. The overall median age was 20.0 (18.0–25.0) years, median BMI was 28.5 kg/m^2^ (25.0–40.4), and median waist circumference was 93.8 cm (81.0–120.0) [12]. The sample was 90% current college student. Overall, 79% of participants were iPhone users while 21% of participants were Android users.

Males had lower self-efficacy for healthy eating (mean score = 92.5, SD = 17.1) when compared to females (mean = 102.3, SD = 13.7, and *p* = 0.02) but there was no gender difference in self-efficacy for exercise (*p* = 0.95). Males had higher consumption of sodium when compared to females (4308 versus 3239 milligrams/day, *p* = 0.01). There were no significant differences across racial subgroups in self-efficacy for healthy eating (*p* = 0.67) or self-efficacy for exercise (*p* = 0.61). There were no significant gender or racial differences in nutrition knowledge scores, although males and whites scored higher on average. Also, there were no significant differences across racial subgroups in healthy eating habits, although whites consumed more added sugars and more sodium than other racial subgroups. See Table 1 for full results.

We also examined the relationship between baseline BMI, self-efficacy for healthy eating and exercise, physical activity, and nutrition habits (Table 2). Higher self-efficacy scores for healthy eating were significantly associated with less total sodium (*r* = −0.37, *p* = 0.007), less saturated fat (*r* = −0.48, *p* < 0.001), and greater fruit consumption (*r* = 0.284, *p* = 0.04). Self-efficacy for exercise was associated with 7-day exercise habits (*r* = 0.280, *p* = 0.03). There was no significant association between self-efficacy and total fiber intake, total vegetable intake, or added sugar intake.

There was an association between fiber intake and other eating habits. Fiber intake was significantly associated with sodium consumption (*r* = 0.294, *p* = 0.03), fruit serving consumption (*r* = 0.487, *p* < 0.001), and vegetable serving consumption (*r* = 0.431, *p* = 0.001). In addition, vegetable serving consumption was associated with fruit serving consumption (*r* = 0.430, *p* = 0.001), and sodium consumption was associated with total saturated fat (*r* = 0.633, *p* < 0.001) and vegetable serving consumption (*r* = 0.405, *p* = 0.003).

BMI was marginally associated with sugar consumption (*r* = 0.29, *p* = 0.04) and fruit consumption (*r* = 0.26, *p* = 0.07) and not associated with healthy eating self-efficacy, exercise self-efficacy, nutrition knowledge, exercise habits, and other healthy eating habits.

## 4. Discussion

The results presented in this paper are from a unique study examining the use of a Smartphone application and text messaging for weight loss in a diverse group of young adults.

Studies report that as many as 68% of first-year college students gain weight [16]. Both males and females have been reported to gain a substantial amount of weight, which significantly increases their BMI and percent body fat over a 4-year period [16]. A recent meta-analysis concluded that weight gain is significant in college students, even beyond the first year, with students gaining up to 20.2 pounds (9.20 kg) over four years [17]. In recent studies reporting a positive relationship between healthy beliefs and behaviors, results tend to be reported as a whole, rather than broken down by gender and race [18, 19]. Therefore, there may be key differences between groups that, if identified, could lead to more successful intervention programs.

Our current study sought to examine results based on gender and race reported. Overall, males had a lower self-efficacy for healthy eating and consumed more sodium on average than females. The lower self-efficacy and subsequent poorer nutritional habits indicate that males may need an intervention that focuses specifically on increasing self-efficacy for healthy eating before researchers can expect to see any significant changes in nutritional habits. It is interesting to note that males scored higher than females on the nutrition knowledge survey in our study. This could indicate that males have the knowledge needed to eat healthy and yet recognize that they cannot commit to healthy eating behaviors, thus leading to lower self-efficacy scores. However, in similar studies conducted in college students, no differences were noted in nutrition knowledge scores between males and females, but reports show that those who score better on nutrition knowledge have healthier eating habits [20, 21]. This information could be used in tailoring interventions in that males could receive less education but more assistance with adherence.

In addition, cultural differences exist that may affect weight perception in late adolescents and young adults. Studies report that black females believe a larger amount of weight is gained normally during college compared to their white counterparts and some identify themselves as normal weight when they are overweight or obese; therefore these characteristics may act to mediate the effects of a weight loss intervention [22, 23]. It is important to note that there is limited research which focuses on race and weight in this population. Of the 49 studies in a recent meta-analysis, only 2 (4%) of the studies were not conducted with predominately Caucasian samples [17]. Of the remaining 47 studies, 40 (85%) had samples comprised of ≥75% Caucasian subjects [17].

Our results from examining racial subgroups by self-efficacy scores indicated no significant differences. Therefore, it appears that the racial groups from the YAWL study had similar beliefs in their ability to perform exercise and healthy eating habits. However, nutritional habits, although not significant, were poorer in whites than other racial subgroups. Other studies conducted had similar results reporting poorer nutrition for whites compared to African Americans and Hispanics [24]. Whites consumed more sodium and added sugars. A more in-depth approach to examining nutrition habits should be performed across racial subgroups. Intervention materials, specifically text messages focusing on healthy eating, may be better tailored to specific nutrients for certain groups. For example, from the current analysis, it appears that white males should receive messages that focus predominately on sodium consumption. In addition, we were limited by the information we obtained from the ASA24. In the future, it may be beneficial to look at whole foods consumed across racial subgroups to better identify differences in diet that could possibly be related to culture.

Self-efficacy for healthy eating scores was significantly associated with better eating habits. In addition, self-efficacy for exercise was associated with better exercise habits. With these results, it appears that self-efficacy could be a prime target area for interventions leading to healthier lifestyle habits. It may be beneficial for researchers to focus on building self-efficacy of a certain behavior before asking a participant to perform that behavior.

Results from the current analysis could contribute to the refinement of the intervention utilizing a Smartphone application and text messaging for weight loss in diverse young adults by providing more information on how to individualize and personalize pieces of the intervention package. Perhaps the structure of weight loss interventions in the young adult population should begin with a period of efficacy building followed by a period of behavior change to see maximum results. Program(s) with that structure should be evaluated in a subsequent randomized controlled trial.

There were several limitations to this analysis. A small sample size of *N* = 62 individuals was analyzed. In addition, all data collected on diet and exercise was self-report. Finally, this study was conducted on a single campus located on the east coast. A larger randomized controlled trial should be conducted with long-term outcomes to determine if these results are reproducible in a larger, diverse sample. Also, instruments should be used to collect direct data on exercise and eating habits rather than relying on self-report.

## 5. Conclusion

The results from this secondary analysis of the YAWL study indicate that self-efficacy for healthy eating and that for exercise are important factors related to healthy eating and exercise behaviors. Although no racial differences were noted, there were gender differences that should be considered when planning future interventions. A larger randomized controlled trial is needed to evaluate the results from this analysis.

## Figures and Tables

**Table 1:** Mean (SD) by sex and race/ethnicity.

	Male (*n* = 18)	Female (*n* = 44)	*p* value^$^	White (*n* = 24)	Black (*n* = 8)	Asian (*n* = 21)	Other (*n* = 9)	*p* value^¥^
Healthy eating self-efficacy	92.5 (17.1)	102.3 (13.7)	**0.02**	101.3 (16.7)	102.1 (11.1)	96.1 (14.3)	99.8 (17.9)	0.67
Exercise self-efficacy	85.7 (22.5)	85.4 (20.5)	0.95	88.5 (24.7)	85.4 (18.1)	80.4 (19.7)	89.6 (15.5)	0.61
Godin leisure time score	42.1 (24.4)	31.9 (19.2)	0.08	34.0 (24.9)	31.1 (22.2)	36.0 (19.1)	38.1 (17.5)	0.92
Nutrition knowledge	28.8 (4.1)	28.1 (3.6)	0.47	29.3 (4.3)	27.5 (3.5)	27.5 (3.5)	28.4 (2.7)	0.40
Total fiber (g)	13.4 (6.6)	15.4 (10.1)	0.48	17.0 (10.9)	16.9 (11.9)	12.5 (7.7)	13.5 (5.1)	0.49
Total sodium (mg)	4308 (1646)	3239 (1203)	**0.01**	3682 (1411)	3499 (1372)	3509 (1583)	3254 (1197)	0.90
Total saturated fat (g)	33.0 (16.1)	25.4 (14.0)	0.11	26.4 (16.1)	31.1 (21.6)	26.6 (13.7)	29.4 (9.9)	0.85
Vegetable servings/day	1.4 (1.1)	1.3 (1.3)	0.81	1.5 (1.0)	0.6 (0.7)	1.5 (1.5)	1.2 (1.2)	0.39
Fruit servings/day	0.7 (1.0)	1.3 (1.6)	0.24	0.9 (1.0)	1.9 (1.1)	1.0 (2.1)	1.1 (1.0)	0.55
Total added sugar (g)	9.8 (10.3)	11.0 (9.2)	0.70	13.9 (11.1)	10.2 (6.9)	7.0 (7.7)	11.6 (8.5)	0.21

^$^ *t*-test.

^*¥*^ANOVA.

**Table 2:** Correlation matrix (method: Pearson correlation).

Variable	Pearson correlation coefficient (*N* = 62)									
	BMI	Healthy eating self-efficacy	Exercise self-efficacy	Godin leisure time score	Nutrition knowledge	Total fiber (g)	Total sodium (mg)	Total saturated fat	Total vegetable servings per day	Total fruit servings per day
BMI	1.000	—	—	—	—	—	—	—	—	—
Healthy eating self-efficacy	0.002	1.000	—	—	—	—	—	—	—	—
Exercise self-efficacy	−0.069	0.312^*∗*^	1.000	—	—	—	—	—	—	—
Godin leisure time score	−0.074	−0.020	0.280^*∗*^	1.000	—	—	—	—	—	—
Nutrition knowledge	0.019	−0.014	0.170	0.093	1.000	—	—	—	—	—
Total fiber (g)	0.010	0.086	0.236	−0.092	0.249	1.000	—	—	—	—
Total sodium (mg)	−0.013	−0.371^*∗∗*^	−0.196	−0.136	0.133	0.294^*∗*^	1.000	—	—	—
Total saturated fat	−0.060	−0.481^*∗∗∗*^	−0.172	−0.194	0.080	0.248	0.633^*∗∗∗*^	1.000	—	—
Total vegetable servings per day	−0.054	0.080	0.091	−0.017	0.225	0.431^*∗∗∗*^	0.405^*∗∗*^	0.053	1.000	—
Total fruit servings per day	0.256	0.284^*∗*^	0.144	−0.066	0.123	0.487^*∗∗∗*^	0.052	−0.128	0.430^*∗∗*^	1.000
Total added sugars	0.288^*∗*^	−0.101	−0.011	−0.292^*∗*^	−0.074	0.257	0.040	0.188	−0.203	0.032

^*∗*^ *p* < 0.05,  ^*∗∗*^*p* < 0.01, and  ^*∗∗∗*^*p* < 0.001.
